# Wild Chimpanzees Infected with 5 *Plasmodium* Species

**DOI:** 10.3201/eid1612.100424

**Published:** 2010-12

**Authors:** Marco Kaiser, Anna Löwa, Markus Ulrich, Heinz Ellerbrok, Adeelia S. Goffe, Anja Blasse, Zinta Zommers, Emmanuel Couacy-Hymann, Fred Babweteera, Klaus Zuberbühler, Sonja Metzger, Sebastian Geidel, Christophe Boesch, Thomas R. Gillespie, Fabian H. Leendertz

**Affiliations:** Author affiliations: Robert Koch-Institute, Berlin, Germany (M. Kaiser, A. Löwa, H. Ellerbrok, A.S. Goffe, A. Blasse, F.H. Leendertz);; GenExpress GmbH, Berlin (M. Kaiser, M. Ulrich);; University of Oxford, Tubney Abingdon, UK (A.S. Goffe, Z. Zommers);; LANADA/LCPA, Bingerville, Côte d’Ivoire (E. Couacy-Hymann);; Budongo Conservation Field Station, Masindi, Uganda (F. Babweteera, K. Zuberbühler);; University of St. Andrews, St. Andrews, Scotland, UK (K. Zuberbühler);; Max-Planck-Institute for Evolutionary Anthropology, Leipzig, Germany (S. Metzger, S. Geidel, C. Boesch, F.H. Leendertz);; Emory University, Atlanta, Georgia, USA (T.R. Gillespie)

**Keywords:** Wild chimpanzees, Plasmodium, zoonoses, human malaria, parasites, natural diversity, tropical rain forest, dispatch

## Abstract

Data are missing on the diversity of *Plasmodium* spp. infecting apes that live in their natural habitat, with limited possibility of human-mosquito-ape exchange. We surveyed *Plasmodium* spp. diversity in wild chimpanzees living in an undisturbed tropical rainforest habitat and found 5 species: *P. malariae*, *P. vivax,* *P. ovale*, *P. reichenowi*, and *P. gaboni.*

Despite ongoing and, in some regions, escalating morbidity and mortality rates associated with malaria-causing parasites, the evolutionary epidemiology of *Plasmodium* spp. is not well characterized. Classical studies of the blood pathogens of primates have found protozoa resembling human malaria parasites in chimpanzees and gorillas (*1*); however, these studies were limited to microscopy, negating conclusions regarding evolutionary relationships between human and ape parasites. Recent studies that used molecular approaches showed that captive and wild chimpanzees (*Pan troglodytes*) and lowland gorillas (*Gorilla gorilla*), as well as captive bonobos (*Pan paniscus*), harbor parasites broadly related to *P. falciparum* (*2**–**5*); wild and captive gorillas and captive bonobos and chimpanzees are sometimes infected with *P. falciparum* itself (*4**–**6*). Further, captive chimpanzees and bonobos have been shown to have malaria parasites related to human *P. ovale* and *P. malariae* (*6**–**8*); *P. vivax* has been identified in various monkeys and 1 semiwild chimpanzee (*5**,**9*). Recently, *P. knowlesi*, a simian malaria species, became the fifth human-infecting species (*10*), highlighting the possibility of transmission of new *Plasmodium* spp. from wild primates to humans.

## The Study

To investigate the prevalence of different *Plasmodium* spp. in wild great apes living in their natural habitat (tropical rainforests), we analyzed tissue samples from 16 wild West African chimpanzees that died primarily of anthrax or respiratory disease in Taï National Park, Côte d’Ivoire. A generic real-time PCR that detects all known *Plasmodium* spp. was used to test all samples for the parasite. Sequence analysis of the *CytB* gene and small subunit rRNA genes was conducted for real-time PCR–positive samples to determine the strain present; 1,140 bp of the *CytB* gene and 765 bp of the *18S* gene of the *Plasmodium* genome were amplified by classic PCR. Resulting products were sequenced either directly or after cloning for rRNA gene and when initial sequence information showed the possible presence of 2 different species (Table).

**Table:** Tissue and fecal samples from wild chimpanzees examined for *Plasmodium* species, Tai National Park, Cote d’Ivoire, and Budongo Forest, Uganda*

Type of sample and name or species of chimpanzee	Genetic sequence copies/mg tissue	*Plasmodium* species detected	GenBank accession nos.
Necropsy			
Loukoum	530	*P. gaboni*	GU815507 (*CytB*), GU815523 (*18S*)
Noah	50	*P. gaboni*	GU815508 (*CytB*), GU815524 (*18S*)
Orest	2.2 x 10	*P. gaboni*	GU815509 (*CytB*), GU815525 (*18S*)
Candy	65	*P. reichenowi*	GU815510 (*CytB*), GU815526 (*18S*)
Atra	100	*P. reichenowi*	GU815511 (*CytB*)
Louise	160	*P. reichenowi*	GU815512 (*CytB*), GU815527 (*18S*)
EastChip 06	105	*P. reichenowi*, *P. gaboni*	GU815512–13 (*CytB*)
Olduvai	130	*P. reichenowi*, *P. malariae*	GU815514–15 (*CytB*), GU815528–29 (*18S*)
Leo	850	*P. malariae*	GU815516 (*CytB*), GU815530 (*18S*)

Kady	105	*P. ovale*	GU815517 (*CytB*), GU815531 (*18S*)
Sagu	760	*P. vivax*	GU815518 (*CytB*), GU815532 (*18S*)
Dorry	Neg		
Virunga	Neg		
Ophelia	Neg		
Akruba	Neg		
Akwaba	Neg		

Fecal samples, n = 30	Positive qPCR results		
* P. t. verus*	21 (2)	*P. gaboni*	GU815519 (*CytB*)
* P. t. schweinfuthii*	12 (3)	*P. reichenowi P. gaboni*	GU815520–22 (*CytB*)

*All chimpanzees were *Pan troglodytes verus* from Tai except *P. t. schweinfuthii* chimpanzees, which were from Budongo Forest. Parentheses indicate the number of samples for which sequences were obtained and used for phylogenetic tree analyses. Neg, negative; qPCR, quantitative PCR.

**Figure 1:**
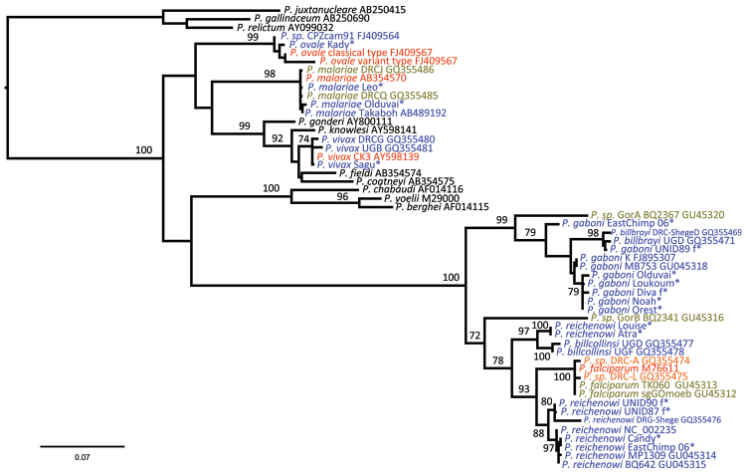
Maximum-likelihood trees of *Plasmodium* spp. obtained from the analysis of a 1,087-bp *CytB* alignment. Blue indicates sequences determined from chimpanzee hosts; green, bonobos; gray, gorillas; and red, humans. Black indicates sequences obtained from nonprimate hosts. *Plasmodium* spp. sequences derived from chimpanzees in this study are marked with an asterisk. Bootstrap values are shown when >70. The tree was rooted using avian plasmodium sequences. Accession numbers of all sequences used are shown in the Table. Scale bar indicates nucleotide substitutions per site.

**Figure 2:**
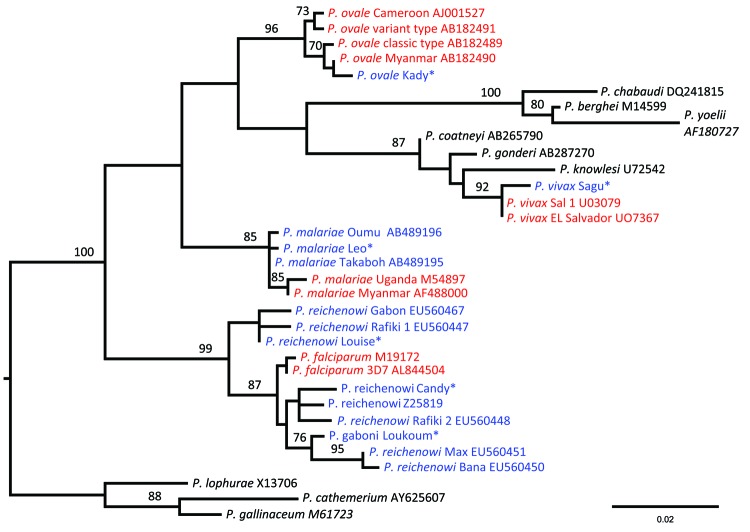
Maximum likelihood tree of *Plasmodium* spp. obtained from the analysis of a 621 bp–long 18S alignment. Blue indicates sequences determined from chimpanzee hosts; green, bonobos; gray, gorillas; and red, humans. Black indicates sequences obtained from nonprimate hosts. *Plasmodium* spp. sequences derived from chimpanzees in this study are marked with an asterisk. Bootstrap values are shown when >70. The tree was rooted using avian plasmodium sequences. Accession numbers of all sequences used are shown in the Table. Scale bar indicates nucleotide substitutions per site.

Phylogenetic analyses of sequences obtained confirmed the presence of 5 species: *P. reichenowi* and *P. gaboni*, which had been found previously (*2**,**3*); but also *P. vivax*, *P. ovale,* and *P. malariae*–like strains (Figure 1, Figure 2). The most prevalent species was *P. reichenowi* (6/16), which had representatives in subclusters *P. gaboni* and *P. reichenowi*. The other species were rare, seen only 1 (*P. ovale* and *P. vivax*) or 2 (*P. malariae*) times. Two chimpanzees showed co-infections with multiple *Plasmodium* spp. (Figure 1, Figure 2), 1 infected with *P. reichenowi* and a *P. malariae*–like strain and the other with *P. reichenowi* and *P. gaboni*.

Is the observed high prevalence of *Plasmodium s*pp. typical for wild chimpanzees or related to reduced immune function associated with the severe infection that was the primary cause of death in each case? To investigate this question, we tested DNA extracted from fecal samples of apparently healthy chimpanzees collected over the past 8 years (n = 30) (*11*) of the same study population by using the generic real-time PCR followed by amplification of the *CytB* gene. Of these samples, 21 (70%) were positive for *Plasmodium* spp. by real-time PCR. Because of low copy numbers in feces, phylogentic analyses were limited to 2 samples in which *P. reichenowi* of the *P. gaboni* subcluster was confirmed.

To determine if the observed high prevalence of plasmodia was a site- or chimpanzee subspecies–specific phenomenon, we tested 30 randomly selected fecal samples of individually known apparently healthy wild Eastern chimpanzees from the Budongo Forest in Uganda. Overall prevalence of *Plasmodium* spp. was lower than in West African chimpanzees but still relatively high (40%); *P. reichenowi* and *P. gaboni* were identified in 3 samples.

Our results demonstrate that the prevalence of different *Plasmodium* spp. in wild chimpanzees is similar to that of untreated human populations in sub-Saharan Africa (www.who.int/malaria). Throughout sub-Saharan Africa, *P. falciparum* is more predominant in humans than are other *Plasmodium* spp. Considering the lack of clinical signs of malaria in chimpanzees from which fecal samples were collected and those that had died of respiratory disease or anthrax, *Plasmodium* spp. infections appear to be asymptomatic or at least nonlethal in wild chimpanzees. However, signs of illness are rarely observed in wild primates because infected animals often mask weakness to maintain social position and avoid attack by predators (*12*). Recently developed technologies for the noninvasive determination of temperature in wild chimpanzees may enable more effective examination of the relationship between the primary clinical feature of malaria (i.e., cyclical fevers) and *Plasmodium* spp. infection (*13*).

*P. ovale* was previously described from captive chimpanzees and *P. malariae* from captive chimpanzees and captive bonobos have been described (*5**–**8*). Our study results demonstrate that *P. malariae* and *P. ovale* occur in wild chimpanzees that inhabit pristine contiguous forest with extremely limited exposure to humans, suggesting the natural existence of these parasites in wild great apes.

Because of a Duffy-negative condition in 95%–99% of the human population in western and central continental Africa, transmission of *P. vivax* does not seem to occur. However, *P. vivax* infections are common in travelers returning from these areas (*11*). Even though we cannot totally exclude the possibility of introduction of *P. vivax* in the chimpanzee population through humans, our discovery of *P. vivax* in wild chimpanzees living exclusively within their natural habitat suggests that wild African apes may be a natural reservoir.

Our study shows the existence of *P. reichenowi* and related strains in wild chimpanzees as described for chimpanzees and gorilla by others (*2**–**4**,**6*). Infections with strains of the *P.* *reichenowi* group (sometimes referred to as the species *P. gaboni*, *P. billbrayi,* and *P. billcollinsi*) appear to occur widely in wild and captive great apes in Africa with some variation between chimpanzee subspecies from biogeographically distinct sites. The wild chimpanzees examined demonstrated no infections with classic human *P. falciparum*. This lack of infection is likely caused by low human presence in their habitat and, consequently, few or no infected vectors, low sample size, or a missing receptor in chimpanzees (*14*). More investigations are needed because recently *P. falciparum* infections have been described for 2 captive chimpanzees (*6*). The situation is clearer for captive and wild lowland gorillas (*Gorilla gorilla*) for which infections and receptors have recently been described (*4*). Infections have also been documented for captive bonobos (*5*).

## Conclusions

Previous examination of the role of our closest phylogenetic relatives, the great apes, in the evolution and persistence of human plasmodia has been limited by a lack of data from wild ape populations where opportunities for human-mosquito-ape malaria exchange are minimal. Interpretation of patterns of malaria infection in captive ape populations, such as sanctuaries and zoos, must consider the ample opportunities for human-to-ape transmission of such parasites, negating the opportunity to investigate the evolutionary origins and public health–related risks of these parasites. Conversely, our examination of these parasites in wild chimpanzees with no contact to the periphery of the rainforest habitat (Technical Appendix Figure ) demonstrates that these apes are most likely naturally infected with *P. ovale*, *P. vivax,* and *P. malariae*, 3 types of plasmodia rarely observed in humans of the region. Whether wild great apes are the origin or reservoirs of these *Plasmodium* types requires further investigation. These results may have implications for global efforts to eradicate malaria in humans, including vaccine development based on animal variants of human parasites.

## Supplementary Material

Technical Appendix Methods used and Sequence Info Used from the Public Database.

## Addendum

While this article was in press, Liu et al. published a study showing strong evidence that *P. falciparum* originated in gorillas (*15*). Their study also recovered other plasmodia, complementing our findings. As recommended in our conclusions section, the Liu et al. study was based on a large number of samples from wild great apes.
