# Supplementary material for: Wild Chimpanzees Infected with 5 Plasmodium Species
Source: Emerg Infect Dis. 2010 Dec;16(12):1956–9. doi: 10.3201/eid1612.100424 (PMC3294549; doi:10.3201/eid1612.100424)
Supplement: Technical Appendix — Methods used and Sequence Info Used from the Public Database. [file 10-0424-Techapp_5p.pdf]

Article DOI: 10.3201/eid1612.100424

Wild Chimpanzees Infected with 5 *Plasmodium* Species

Technical Appendix

Methods

Ethics Statement

No living nonhuman primates were used for this study. Tissue samples were obtained from animals that died of various causes (see sample collection) in the Tai National Park, Côte d'Ivoire. Noninvasive samples (feces) were collected without disturbing animal behavior or interfering with them. Tissue samples were exported from Côte d'Ivoire under permission of the CITES authorities and fecal samples were exported according to the regulations of each country.

Sample Collection

Spleen samples of wild chimpanzees that died of anthrax or respiratory disease in Tai National Park, Côte d'Ivoire were collected using single-use equipment and appropriate safety measures (*1*–*4*). In addition, over a period of 8 years, fecal samples were collected from known living individuals of the same chimpanzee population and within a 12-month period in 2007 from individually known wild chimpanzees of various communities living in the Budongo Forest area, Uganda. Samples were collected using single-use gloves avoiding any contamination with human pathogens. All samples from Taï National Park were preserved in liquid nitrogen. Samples from Budongo Forest were preserved in RNAlater (QIAGEN, Hilden, Germany) and later frozen.

DNA Extraction, Amplification, and Sequencing

DNA was extracted from the spleen of 16 chimpanzees using the DNeasy Blood and Tissue Kit (QIAGEN). DNA was extracted from 30 fecal samples from each of the chimpanzee sites by using the EURx GeneMATRIX Stool DNA Purification Kit (Roboklon, Berlin, Germany). An in-house real time qPCR, P.sp. 18S F (5´-gTT TCT gAC CTA TCA gCT TTT gAT gT-3´), P.sp. 18S R1 (5´-CTg CCT TCC TTA gAT gTg gTA CCT A-3´) and

P.sp. TM (5´-YAK-CAg gCT CCC TCT CCg gAA TCg AAC-BBQ-3´), was used to analyze samples for the presence of *Plasmodium* spp. Each sample was analyzed in duplicate. The detection limit was 10 copies of target DNA per reaction. qPCR positive samples were used for classic PCRs aiming at amplifying 1140bp of cytB gen and 765bp fragment of the small subunit rRNA gen for phylogenetic analysis.

For each PCR reaction, 5 µL 10 x buffer, 3µL $MgCl_2$ 50mM, 4 µL dNTPs 10 mM, 1 µL each primer 10 µM, 0.5 µL (2.5 U) Platinum Taq (Invitrogen, Carlsbad, CA, USA) and 5 µL DNA were used. Reaction volume was adjusted with water (Molecular Biology Grade Eppendorf) to 50µL. The reaction was run in a thermocycler (Mastercycler epgradient, Eppendorf) at 95°C for 2 min, 10 cycles at 95 °C for 15 sec, at 68 °C for 15 s and at 72 °C for 70 s with reducing the annealing temperature 1 ° /cycle and 35 cycles at 95 °C for 15 sec, at 58 °C for 15 s, and at 72 °C for 70 s. The final extension was 72 °C for 5 min.

The following primers were used: P.sp. CytB F (5´-TgC CTA gAC gTA TTC CTg ATT ATC CAg-3´) and P.sp. CytB R (5´-CTT gTg gTA ATT gAC ATC CWA TCC-3´) to amplify cytB gene and P.sp. 18S F (5´-gTT TCT gAC CTA TCA gCT TTT gAT gT-3´) and P.sp. 18S R (5´-TCT gAT CgT CTT CAC TCC CTT AAC-3´) to amplify 18S gene. PCR products were separated by 1.5% agarose gel electrophoresis, purified with NucleoSpin Extract II (Macherey-Nagel, Düren, Germany) and sequenced with the ABI PRISM Big Dye Terminator cycle sequencing kit, according to the manufacturer’s protocol. Sequences were determined using the ABI 310 automated DNA sequencer and analyzed with the ABI PRISM DNA Sequencing Analysis Software (Version 3.7, Applied Biosystems).

Phylogenetic Analysis

Plasmodium sequences determined in this investigation were compared with published sequences using the BLAST network program from the National Center for Biotechnology Information (NCBI). CytB and 18S sequences were aligned to a selection of published sequences using multiple sequence alignment (MUSCLE) (*5*) as implemented in SeaView (*6*) and T-Coffee, which was run on a dedicated webserver (www.tcoffee.org). Conserved blocks were selected from the best alignment (as determined by visual inspection) by using Gblocks (*7*), which was run on the Gblocks webserver (http://molevol.cmima.csic.es/castresana/Gblocks_server.html). The resulting alignments were haplotyped by using Fabox. The overall process came to generating a 1087bp-long CytB alignment comprising 54 taxa and a 621bp-long 18S alignment composed of 33 taxa, which served as input for further analyses. Likelihoods of models of evolution (JC, HKY and GTR;

+F; +I, +G, +I+G) were estimated and then compared according to the Akaike information criterion by using jModelTest (*8*). The models of evolution to which the datasets were a better fit were GTR+I+G for CytB and GTR+G for 18S.

Maximum likelihood (ML) phylogenetic trees were finally estimated under these models using PhyML (*9*) as implemented on a dedicated webserver (http://www.atgc-montpellier.fr/phyml/). Equilibrium frequencies, topology, and branch lengths were optimized and the tree search was realized by using a combination of hill-climbing algorithms (NNI & SPR). Branch robustness was assessed by nonparametric bootstrapping (500 pseudo-replicates).

Sequence Info Used from the Public Database

For CytB: M76611, FJ409566, FJ409567, AB354570, AY598139, GU045312 - GU045316, GU045318, GU045320, GQ355469, GQ355471, GQ355474 – GQ355478, GQ355480, GQ355481, GQ355485, GQ355486, NC_002235, FJ895307, FJ409564, AB489192, AY598141, AB354574, AB354575, AY800111, M29000, AF014115, AF014116, AB250415, AB250690, AY099032.

For 18S: M19172, AL844504, U07367, U03079, AB182489 - AB182491, AJ001527, M54897, AF488000, Z25819, EU560447, EU560448, EU560450, EU560451, EU560467, U72542, AB265790, AB287270, AB489195, AB489196, M14599, AF180727, DQ241815, X13706, M61723, AY625607.

References

1. Leendertz FH, Ellerbrok H, Boesch C, Couacy-Hymann E, Mätz-Rensing K, Hackenbeck R, et al. Anthrax kills wild chimpanzees in a tropical rainforest. Nature. 2004;430:451–2. PubMed DOI: 10.1038/nature02722

2. Köndgen S, Kühl H, N’Goran PK, Walsh PD, Schenk S, Ernst N, et al. Pandemic human viruses cause decline of endangered great apes. Curr Biol. 2008;18:260–4. PubMed DOI: 10.1016/j.cub.2008.01.012

3. Leendertz FH, Pauli G, Maetz-Rensing K, Boardman W, Nunn C, Ellerbrok H, et al. Pathogens as drivers of population declines: the importance of systematic monitoring in great apes and other threatened mammals. Biol Conserv. 2006;131:325–37. DOI: 10.1016/j.biocon.2006.05.002

4. Gillespie TR, Nunn CL, Leendertz FH. Integrative approaches to the study of primate infectious disease: implications for biodiversity conservation and global health. Am J Phys Anthropol. 2008137:53–69. PubMed DOI: 10.1002/ajpa.20949

5. Edgar RC. MUSCLE: multiple sequence alignment with high accuracy and high throughput. Nucleic Acids Res. 2004 Mar 19;32(5):1792–7.

6. Gouy M, Guindon S, Gascuel O. SeaView version 4: a multiplatform graphical user interface for sequence alignment and phylogenetic tree building. Mol Biol Evol. 2010 Feb;27(2):221–4.

7. Castresana J. Selection of conserved blocks from multiple alignments for their use in phylogenetic analysis. Mol Biol Evol. 2000 Apr;17(4):540–52.

8. Posada D. jModelTest: phylogenetic model averaging. Mol Biol Evol. 2008 Jul;25(7):1253–6. Epub 2008 Apr 8.

9. Guindon S, Gascuel O. A simple, fast, and accurate algorithm to estimate large phylogenies by maximum likelihood. Syst Biol. 2003 Oct;52(5):696–704.

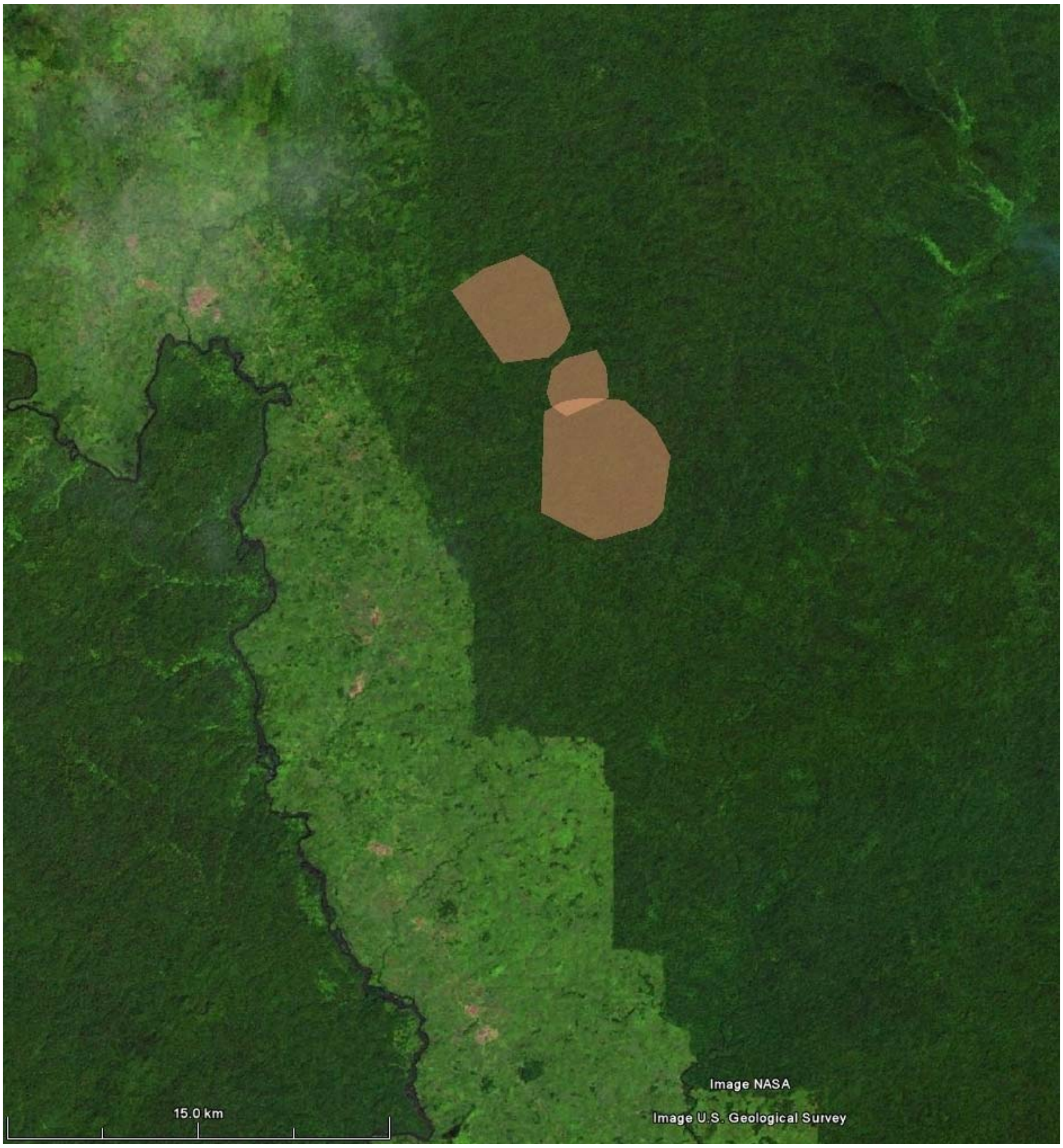

Technical Appendix Figure. Satellite image of habitat of wild chimpanzees investigated in Taï National Park, Côte d'Ivoire. The shaded areas show the maximum extension of the territories of the communities studied. Pristine forest is dark green.
